# Quality of life and its association with psychiatric symptoms and socio-demographic characteristics among people with schizophrenia: A hospital-based cross-sectional study

**DOI:** 10.1371/journal.pone.0229514

**Published:** 2020-02-24

**Authors:** Defaru Desalegn, Shimelis Girma, Tilahun Abdeta

**Affiliations:** 1 Department of Psychiatry, Mettu University, Mettu, Ethiopia; 2 Department of Psychiatry, Institute of Health, Jimma University, Jimma, Ethiopia; 3 Department of Psychiatry, School of Nursing and Midwifery, College of Health and Medical Sciences, Haramaya University, Harar, Ethiopia; St. Paul's Hospital Millenium Medical College, ETHIOPIA

## Abstract

**Objectives:**

To identify sociodemographic and illness-related factors associated with quality of life among people with Schizophrenia.

**Methods:**

A hospital-based cross-sectional study design was employed among 351 people with schizophrenia and attending the followup service at Jimma University Medical Center, psychiatric clinic during the study period. Participants were recruited using a systematic random sampling method and a sample fraction of two was used after the first person was identified by a lottery method. Data entry was done using EpiData version 3.1 and then exported to Statistical Package for Social Sciences version 25 for analysis. Multiple regression analysis was used to determine the statistically significant association between quality of life and independent variables.

**Results:**

Among the four domains of quality of life, respondents scored the lowest mean in the social relationships domain (10.14 ± 3.12). Final adjusted multiple regression model revealed, being divorced was negatively associated with the physical domain (*β* = −0.72, *p* = 0.02), having no formal education was negatively associated with physical health domain (*β* = −0.69, *p* = 0.001) and age was positively associated with the psychological domain (*β* = 0.371, *p* = 0.071). Being rural resident was negatively associated with physical domain (*β* = −0.48, *p* = 0.01), with environmental domain (*β* = −0.64, *p* = 0.03), with social relationships domain (*β* = −0.45, *p* = 0.04), and with overall quality of life (*β* = −1.93, *p* = 0.006). Positive symptoms (*β* = −0.22, *p* = 0.001), negative symptoms (*β* = −0.36, *p* = 0.001), and general psychopathology (*β* = −0.098, *p* = 0.006) were inversely associated with overall quality of life.

**Conclusion:**

In this study, the social relationship domain of quality of life among people with schizophrenia has the lowest mean score. Some socio-demographic variables and psychiatric symptoms were found to be key significant associated factors of quality of life. Priority interventions to improve the social deficits and addressing psychiatric symptoms of people with schizophrenia is essential to improve their quality of life.

## Introduction

Schizophrenia is one of the most severe, chronic and disabling mental disorders[1]. It affects general health, functioning, autonomy, subjective wellbeing, and individuals' perception of reality[2]. Globally, as estimated by the World Health Organization (WHO), about 24 million people suffer from schizophrenia [3]. It was ranked as one of the top ten illnesses contributing to the global burden of disease [4]. The course of schizophrenia appears to be favorable in about 20% of people with schizophrenia, and a small number of individuals are reported to recover completely [5]. There was a shift in the concept of treatment with more emphasis on patient's perspectives like the quality of life [6]. Schizophrenia affects many areas of functioning; people with the illness often lead an isolated and marginalized existence in poor housing, with a low income, little education, and poor vocational and social skills [2]. Most individuals are employed at a lower level and the majority of them have limited social contacts outside of their family [5]. Patients with schizophrenia are also prone to stigma, which leads to discrimination and thus affects their life opportunities and quality of life [6].

“Quality of life (QoL) among individuals with schizophrenia has been measured from both subjective and objective points of view. Subjective measures of QoL include general indicators of life satisfaction and several life domains such as satisfaction with work, family, social relations, finances, and housing situations”. The objective measures of QoL usually include indicators of external life conditions, socio-demographic items, and a functioning role in society [7]. However, the World Health Organization (WHO) focused on subjective aspect and defines the quality of life as “individuals' perception of their position in life in the context of the culture and value systems with their goals, expectations, standards and concerns” [8].

A study done in China found that people with schizophrenia who were treated in primary care had a lower level of QoL when compared to the general population [9]. A study done in Bangladesh found out, most of the people with schizophrenia lead poor to moderate quality of life [10]. Quality of life among people with schizophrenia can be affected by various factors like socio-demographic variables, the severity of psychiatric symptoms, duration of untreated illness, duration of treatment [10, 11], social support, substance use disorder and so on [12].

A study done in Spain among people with schizophrenia found that young people, women, married persons, and those with a low level of education report better quality of life [13]. Also, a study from Jordan reveals that QoL among people with schizophrenia was correlated positively with social support, patients' educational, income level, and employment [11]. However, one study done in Sweden showed that the socio-demographic variables have a weak influence on the patient’s self-assessed quality of life [14]. Study done on psychiatric symptoms and quality of life of people with schizophrenia found that positive and negative symptoms were more strongly related to poor QoL, whereas general psychopathology showed a consistent negative relationship with QoL [15] and another study found out quality of life is negatively correlated with negative symptoms and general psychopathology [16]. Assessing the determinant factors of quality of life of people with schizophrenia is particularly important to minimize the impact of the disorder and improve their quality of life. In Ethiopia, only limited national wide studies conducted on the quality of life and associated factors among people with schizophrenia and there is no study conducted specifically in the study area. Even the available studies in Ethiopia did not try to see the association between quality of life and psychiatric symptoms of people with schizophrenia [17]. Therefore, this study aimed to assess the profile of quality of life and its association with psychiatric symptoms and socio-demographic variables among people with schizophrenia and attending follow up service at Jimma University Medical Center, psychiatry clinic, Southwest Ethiopia.

## Materials and methods

### Study setting

The study was conducted from April 15—June 15, 2018, at Jimma University Medical Center (JUMC) which is one of the oldest governmental hospitals, established in 1937 during the Italian occupation for the service of their soldiers. The psychiatric clinic of JUMC was established in 1988 and gives clinical service for about 15 million population with a total of 40 beds for in-patient service and seven Out Patient Department (OPD) in Southwest Ethiopia [18]. Currently, there are more than 1000 patients with schizophrenia who are attending follow up service at OPD monthly and on average, about 50 patients are visiting psychiatric clinics daily.

### Study design

The hospital-based cross-sectional study design was employed.

### Source population

All patients with schizophrenia and who are attending follow up service at Jimma University Medical Center, psychiatric clinic from April 15—June 15, 2018.

### Study population

Sampled study participants diagnosed with schizophrenia and who attended the follow-up service at Jimma University Medical Center, psychiatric clinic during the study period. Three hundred fifty-one adult persons (age 18 and above) who were diagnosed with schizophrenia according to the diagnostic criteria of the Diagnostic Statistical Manual (DSM-IV or DSM-5) were included in the study. Patients whose acute episode symptoms pose difficulty to communicate were excluded from the study.

### Sample size determination and sampling technique

The minimum sample size required for this study was determined by using the formula to estimate single population mean; using the assumptions of margin of error (d = 1); standard deviation of mean quality of life score 9.13 as reported by previous related study [14]; Z—value at (∞ = 0.05) to be 1.96 and adding 10% non-response rate.

$${Minimum\;sample\;size\;(n)=\frac{{\left(\frac{Z\alpha }{2}\right)}^{2\;}\delta }{d^{2}}}^{2}$$

The total sample size was 352. Study participants were recruited using a systematic random sampling method and a sample fraction of two was used after the first person was identified by a lottery method. We used patients' unique identification card as a questionnaire code to avoid repeated inclusion of patients.

### Data collection instrument and technique

Data was collected by face to face interviews using a semi-structured and pre-tested questionnaire. Data collection tools were prepared first in English and translated into local languages (Afaan Oromo and Amharic) then re-translated back to English by another person who was blinded for the English version to check the clarity of the questionnaire. The questionnaire comprised 3 parts. The first part was socio-demographic variables which include age, sex, ethnicity, religion, marital status, educational status, occupational status, and income. These sociodemographic characteristics were adapted from previous studies. The second part of our questionnaire was psychiatric symptoms including (positive symptoms, negative symptoms, and general psychopathology) and assessed by Positive and negative syndrome scale (PANSS) [19]. The third part was the quality of life of people with schizophrenia which was assessed by the World Health Organization Quality of Life Scale-Brief version (WHOQoL-BREF) [20]. WHOQoL-BREF comprises 26 items self-administered generic questionnaire and it is a short version of the WHO QoL-100 scale [20, 21]. This tool is a sound, cross-culturally valid assessment of QoL [22] and it is found a suitable tool for the assessment of QoL for the person with schizophrenia [23]. WHOQOL-BREF was found to have good reliability (a high internal consistency) and validity which grants the suitability of the tool for a person with schizophrenia [23]. The tool has four domain scores: physical health (7 items), psychological health (6 items), social relationships (3 items), environmental health domain (8 items) as well as two separately scored items about the individuals’ perception of their quality of life (QI) and health (Q2). The scores of each item in each domain were added, in case of negative items (i.e. the response of Q3, Q4, and Q26 were reverse coded) and multiplied by 4 to be directly comparable with WHOQOL-100. Where more than 20% of the data is missing from an assessment, the assessment should be discarded. Domain scores are scaled in a positive direction (i.e. higher scores correspond to a better quality of life). The WHOQoL-BREF scale in this study demonstrated a high internal consistency reliability coefficient (Cronbach's alpha = 0.96). Positive and negative syndrome scale (PANSS) has 30 items which was rated on 7 point Likert scale (Absent = 1, minimal = 2, mild = 3, moderate = 4, moderate severe = 5, severe = 6 and extreme = 7). The scale was conceived as a carefully defined and operationalized method that evaluates positive, negative, or other symptom dimensions based on a formal semi-structured clinical interview and other informational sources. The items are grouped into three; 7 are grouped to form a positive scale, measuring symptoms that are superadded to normal mental status, and 7 items constitute negative scale, assessing features absent from normal mental status, remaining 16 items constitute general psychopathology scale that measures the general psychiatric symptoms [19]. The psychometric properties of PANSS indicated that the positive scale and negative scale sub-item had high internal consistency [24]. For data collection, four Master of Science in integrated clinical and community mental health students were recruited. Training on the data collection tool, on study objectives, on data collection methods, and ethical issues were given for data collectors and supervisors for one day. The pre-test was conducted on 5% of the sample of people with schizophrenia and attending follow up service at Agaro general hospital, 45 km from Jimma. Data collectors were supervised by two Masters of Science students in public health and the principal investigators. On each day of data collection, the completed questionnaires were checked for completeness and finally, the collected data were entered into a computer.

### Data processing and analysis

Collected data were checked, coded and entered into EpiData Version 3.1 and then exported to Statistical Package for Social Science Version 25.0 for further analysis. Descriptive statistics, such as frequency, mean and standard deviations were computed. Linearity assumptions were checked by scatter plot, homogeneity of variances was checked by scatter plots, and there was no heteroscedasticity/no clear pattern on scatter plot. Skewness that ranged in between ±1 was taken as normally distributed and all variables satisfied normality assumptions. Multicollinearity was checked and the minimum and maximum variable inflation factors reported were 1.025 and 4.553 respectively, which indicates that there was no multicollinearity threat. Bivariate/simple linear regression analysis was done for each independent variable against the dependent variable and variables with p-value < 0.25 were considered as a candidate for multiple regression. In the final adjusted multiple regression, variables with a *P*-value <0.05 were declared significantly associated with quality of life. Un-standardized Beta (β) coefficients with 95% confidence interval (CI) were computed to assess the level of association and statistical significance in multiple regression analysis.

### Data quality control

One day training was given for data collectors and supervisors. The pre-test was conducted among people with schizophrenia of another hospital to identify potential problems on data collection tools and to check the consistency of the questionnaire and modification of the questionnaire. Regular supervision and support were given for data collectors by the supervisors and principal investigator to ensure that all necessary data are properly collected. The collected data were checked for completeness and consistency by supervisors and principal investigators on a daily bases during data collection time.

### Ethical consideration

Ethical clearance was obtained from the Research Ethical Review Board of Jimma University, institute of health and the study was performed according to the declaration of Helsinki. Written informed consent was obtained from study participants. Participants were told the right to refuse or discontinue participation at any time they want and the chance to ask anything about the study. After data entry was completed, the questionnaires were kept securely locked.

## Results

### Socio-demographic characteristics of the study participants

The mean age of participants was 33 years (*SD* ± 8) ranging from 18 to 54 years. More than half of the study participants were married (*n* = 193, 55%) and around one-third was single (*n* = 120, 34.2%). Majority of study participants (*n* = 242, 68.9%) were male and (*n* = 203, 57.8%) were urban resident. Regarding their educational status, (*n* = 99, 28.2%) of study participants were attended primary school (Table 1).

**Table 1:** Socio-demographic characteristics of people with schizophrenia (*n* = 351).

Variables	Variable categories	Frequency(n)	Percentage(%)
**Sex**	Male	242	68.9
	Female	109	31.1
**Marital status**	Single	120	34.2
	Married	193	55.0
	Divorced	30	8.5
	Widowed	8	2.3
**Religion**	Muslim	228	65.0
	Orthodox Christian	66	18.8
	Protestant Christian	57	16.2
**Ethnicity**	Oromo	236	67.2
	Amhara	38	10.8
	Yem	6	1.7
	Dawuro	13	3.7
	Kaffa	30	8.5
	Other*	28	8.0
**Educational status**	No formal education	95	27.1
	Grade 1–8	99	28.2
	Grade 9–10	77	21.9
	Diploma and above	80	22.8
**Residence**	Urban	203	57.8
	Rural	148	42.2
**Age**	< = 33	179	51.0
	>33	172	49.0
**Occupational status**	Government worker	79	22.5
	Farmer	84	23.9
	Merchant	35	10.0
	Housewife	45	12.8
	Daily labor	45	12.8
	Students	63	17.9

Other*- Tigre, Walayta

### Perceived quality of life among people with schizophrenia and attending the follow-up service at Jimma University Medical Center, psychiatric clinic

In this study, the respondents’ mean (SD)score of their overall quality of life was 44.81 ± 8.87 and 51.3% (*n* = 180) and 48.7% (*n* = 171) of the study participants scored below the mean and above the mean in overall quality of life respectively. The minimum and maximum mean scores of the study participants in overall quality of life were 19 and 63 respectively. Among the four domains of quality of life, respondents scored highest mean in the physical health domain (12.11 ± 2.04) and scored lowest mean in the social relationship domain (10.14 ± 3.12) respectively (Table 2).

**Table 2:** Profile of quality of life among people with schizophrenia and attending the follow-up service at Jimma University Medical Center, psychiatric clinic (*n* = 351).

Domains	Mean (± SD)	Range	
		Minimum	Maximum
Physical Health	12.11 ± 2.0	4	19
Psychological Health	11.14 ± 2.26	5	17
Social Relationships	10.14 ± 3.12	4	20
Environmental Health	11.42 ± 2.60	5	17
Overall Quality of life	44.81 ± 8.87	19	63

### Self-rated perceived quality of life and health satisfaction among people with schizophrenia and attending the follow-up service at Jimma University Medical Center, psychiatric clinic

The study participants were asked to provide their perception of their quality of life and health satisfaction. Concerning their perceived quality of life, more than one-third 39.0% (*n* = 137) respondents reported that their perceived quality of life was poor and followed by 31.9% (*n* = 112) who reported neither poor nor good. Based on their perceived satisfaction of their health, 38.5% (*n* = 135) participants were dissatisfied and 2.6% (n = 9) were very satisfied (Table 3).

**Table 3:** Self-rated perceived quality of life and health satisfaction among people with schizophrenia (*n* = 351).

	Frequency (n = 351)	Percent
**Perceived quality of life**		
Very poor	15	4.3
Poor	137	39.0
Neither poor nor good	112	31.9
Good	82	23.4
Very good	5	1.4
**Perceived health satisfaction**		
Very dissatisfied	12	3.4
Dissatisfied	135	38.5
Neither dissatisfied nor satisfied	101	28.8
Satisfied	94	26.8
Very satisfied	9	2.6

### Psychiatric symptoms among people with schizophrenia and attending the follow-up service at Jimma University Medical Center, psychiatric clinic

Among the psychiatric symptoms, the study participants scored low and high mean score in positive symptoms (15.21 ± 9.28) and general psychopathology (30.26 ± 18.13) respectively (Table 4).

**Table 4:** Profile of psychiatric symptoms among people with schizophrenia and attending the follow-up service at Jimma University Medical Center, psychiatric clinic (*n* = 351).

Variables	Mean (± SD)	Range	
		Minimum	Maximum
Positive symptom	15.21 ± 9.28	7	38
Negative symptom	18.22 ± 9.52	7	40
General psychopathology	30.26 ± 18.13	16	81

### Factors associated with quality of life among people with schizophrenia and attending the follow-up service at Jimma University Medical Center, psychiatric clinic

We performed simple and multiple regression analyses to identify factors associated with the respondents’ various domains of quality of life and overall quality of life. The result of adjusted multiple regression analysis fitted for the physical health domain showed, about 41.5% of the total variation in the physical health domain was explained by variables in the model (*Adjusted R*2 = 0.415, *p* = 0.001). Being divorced (*β* = −0.72, *p* = 0.02), having no formal education (*β* = −0.69, *p* = 0.001), being rural resident (*β* = −0.48, *p* = 0.01), positive symptoms (*β* = −0.07, *p* = 0.001), and negative symptoms (*β* = −0.04, *p* = 0.02) were inversely associated with physical health domain. About 46.6% of the total variation in the psychological domain was explained by variables in the model (*Adjusted R*2 = 0.466, *p* = 0.001). Positive symptoms (*β* = −0.05, *p* = 0.004) and negative symptoms (*β* = −0.11, *p* = 0.001) were inversely associated with the psychological domain of quality of life among people with schizophrenia, while age (*β* = 0.371, *p* = 0.071) was positively associated with the psychological domain (Table 5).

**Table 5:** Adjusted multiple regression analysis models showing independently associated factors with domains of quality of life and overall quality of life among people with schizophrenia (*n* = 351).

Quality of life and its domains	Unstandardized coefficient (β)	(95% *CI for β*)	P-value	Multicollinearity statistics	
				Tolerance	VIF
**Physical health domain (*n* = 351)**					
**Sex**					
Male	0.028	(-0.348 to 0.404)	0.885	0.860	1.163
Female	-0.028	(-0.404 to 0.348)	0.885	0.860	1.163
**Marital status**					
Single	-0.022	(-0.392 to 0.348)	0.907	0.847	1.181
Married	0.11	(-0.32 to 0.53)	0.63	0.849	1.19
Divorced	-0.72	(-1.333 to -0.101)	0.02	0.878	1.139
Widowed	0.795	(-0.340 to 1.930)	0.169	0.907	1.102
**Educational status**					
No formal education	-0.63	(12.03 to 12.52)	0.001	0.891	1.11
Grade 1–8	0.470	(0.001 to 0.938)	0.114	0.586	1.707
Grade 9–10	0.778	(0.268 to 1.289)	0.113	0.584	1.714
Diploma and above	0.419	(-0.103 to 0.940)	0.115	0.545	1.836
**Residence**					
Urban	0.481	(0.107 to 0.856)	0.01	0.762	1.313
Rural	-0.481	(-0.856 to -0.107)	0.01	0.762	1.313
**Age of respondents**	-0.139	(-0.466 to 0.187)	0.402	0.975	1.025
**Psychiatric symptoms**					
Positive symptoms	-0.070	(-0.101 to -0.039)	0.001	0.314	3.185
Negative symptoms	-0.043	(-0.078 to -0.008)	0.02	0.236	4.229
General psychopathology	-0.015	(-0.034 to 0.004)	0.130	0.220	4.553
**Psychological domain (*n* = 351)**					
**Sex**					
Male	0.253	(-0.152 to 0.657)	0.220	0.860	1.163
Female	-0.253	(-0.657 to 0.152)	0.220	0.860	1.163
**Marital status**					
Single	-0.060	(-0.458 to 0.338)	0.768	0.847	1.181
Married	0.155	(-0.323 to 0.634)	0.523	0.849	1.19
Divorced	-0.113	(-0.776 to 0.550)	0.737	0.878	1.139
Widowed	0.454	(-0.768 to 1.676)	0.465	0.907	1.102
**Educational status**					
No formal education	-0.210	(-0.745 to 0.326)	0.442	0.891	1.11
Grade 1–8	0.153	(-0.351 to 0.657)	0.551	0.586	1.707
Grade 9–10	0.206	(-0.343 to 0.756)	0.460	0.584	1.714
Diploma and above	-0.019	(-0.580 to 0.542)	0.947	0.545	1.836
**Residence**					
Urban	0.371	(-0.032 to 0.774)	0.071	0.762	1.313
Rural	-0.371	(-0.774 to 0.032)	0.071	0.762	1.313
**Age of respondents**	0.371	(-0.032 to 0.774)	0.071	0.762	1.313
**Psychiatric symptoms**					
Positive symptoms	-0.05	(-0.083 to -0.016)	0.004	0.314	3.185
Negative symptoms	-0.11	(-0.146 to -0.071)	0.001	0.236	4.229
General psychopathology	-0.009	(-0.029 to 0.012)	0.399	0.220	4.553
**Environmental domain (*n* = 351)**					
**Sex**					
Male	0.197	(-0.362 to 0.756)	0.489	0.860	1.163
Female	-0.197	(-0.756 to 0.362)	0.489	0.860	1.163
**Marital status**					
Single	-0.022	(-0.571 to 0.527)	0.937	0.847	1.181
Married	0.081	(-0.579 to 0.742)	0.809	0.849	1.19
Divorced	-0.669	(-1.584 to 0.246)	0.151	0.878	1.139
Widowed	1.376	(-0.311 to 3.062)	0.110	0.907	1.102
**Educational status**					
No formal education	-0.494	(-1.232 to 0.243)	0.188	0.891	1.11
Grade 1–8	0.434	(-0.262 to 1.129)	0.221	0.586	1.707
Grade 9–10	0.555	(-0.203 to 1.314)	0.151	0.584	1.714
Diploma and above	0.054	(-0.720 to 0.829)	0.890	0.545	1.836
**Residence**					
Urban	0.635	(0.079 to 1.191)	0.03	0.762	1.313
Rural	-0.64	(-1.191 to -0.079)	0.03	0.762	1.313
**Age of respondents**	0.052	(-0.433 to 0.538)	0.832	0.975	1.025
**Psychiatric symptoms**					
Positive symptoms	-0.019	(-0.065 to 0.027)	0.421	0.314	3.185
Negative symptoms	-0.091	(-0.143 to -0.039)	0.001	0.236	4.229
General psychopathology	-0.07	(-0.093 to -0.037)	0.001	0.220	4.553
**Social relationship domain (*n* = 351)**					
**Sex**					
Male	0.122	(-0.307 to 0.550)	0.576	0.860	1.163
Female	-0.122	(-0.550 to 0.307)	0.576	0.860	1.163
**Marital status**					
Single	-0.253	(-0.675 to 0.168)	0.237	0.847	1.181
Married	0.393	(-0.155 to 0.941)	0.159	0.849	1.19
Divorced	-0.454	(-1.156 to 0.248)	0.204	0.878	1.139
Widowed	0.733	(-0.560 to 2.027)	0.266	0.907	1.102
**Educational status**					
No formal education	-0.127	(-0.742 to 0.488)	0.685	0.891	1.11
Grade 1–8	0.160	(-0.374 to 0.693)	0.557	0.586	1.707
Grade 9–10	0.359	(-0.223 to 0.940)	0.226	0.584	1.714
Diploma and above	-0.113	(-0.707 to 0.481)	0.710	0.545	1.836
**Residence**					
Urban	0.446	(0.019 to 0.873)	0.041	0.762	1.313
Rural	-0.45	(-0.873 to -0.019)	0.04	0.762	1.313
**Age of respondents**	0.141	(-0.232 to 0.513)	0.457	0.975	1.025
**Psychiatric symptoms**					
Positive symptoms	-0.08	(-0.113 to -0.043)	0.001	0.314	3.185
Negative symptoms	-0.12	(-0.157 to -0.078)	0.001	0.236	4.229
General psychopathology	-0.010	(-0.032 to 0.011)	0.355	0.220	4.553
**Overall quality of life (*n* = 351)**					
**Sex**					
Male	0.599	(-0.776 to 1.974)	0.392	0.860	1.163
Female	-0.599	(-1.974 to 0.776)	0.392	0.860	1.163
**Marital status**					
Single	-0.357	(-1.709 to 0.994)	0.604	0.847	1.181
Married	0.735	(-1.138 to 2.608)	0.441	0.849	1.19
Divorced	-1.953	(-4.205 to 0.300)	0.089	0.878	1.139
Widowed	3.358	(-0.792 to 7.509)	0.112	0.907	1.102
**Educational status**					
No formal education	-1.456	(-3.549 to 0.638)	0.172	0.891	1.11
Grade 1–8	1.216	(-0.497 to 2.929)	0.164	0.586	1.707
Grade 9–10	1.898	(0.032 to 3.765)	0.046	0.584	1.714
Diploma and above	0.341	(-1.565 to 2.247)	0.725	0.545	1.836
**Residence**					
Urban	1.93	(0.564 to 3.303)	0.006	0.762	1.313
Rural	-1.93	(-3.303 to -0.564)	0.006	0.762	1.313
**Age of respondents**	-0.036	(-1.231 to 1.159)	0.953	0.975	1.025
**Psychiatric symptoms**					
Positive symptoms	-0.22	(-0.330 to -0.103)	0.001	0.314	3.185
Negative symptoms	-0.36	(-0.487 to -0.232)	0.001	0.236	4.229
General psychopathology	-0.098	(-0.168 to -0.029)	0.006	0.220	4.553

Regarding environmental domain, about 46.6% of the total variation in the domain was explained by variables in the model (*Adjusted R*2 = 0.466, *p* = 0.001) and variables including being rural resident (*β* = −0.64, *p* = 0.03), negative symptoms (*β* = −0.09, *p* = 0.001) and general psychopathology (*β* = −0.07, *p* = 0.001) were inversely associated with environmental domain. Also, about 54.7% of the total variation in social relationship domain of quality of life among people with schizophrenia was explained by variables in the model (*Adjusted R*2 = 0.547, *p* = 0.001 and being rural resident (*β* = −0.45, *p* = 0.04), positive symptoms (*β* = −0.08, *p* = 0.001), and negative symptoms (*β* = −0.12, *p* = 0.001) were inversely associated with social relationship domain of participants (Table 5).

Concerning the overall quality of life of people with schizophrenia, about 59.9% of the total variation in overall quality of life was explained by variables in the model (*Adjusted R*2 = 0.599, *p* = 0.001 and variables including being rural resident (*β* = −1.93, *p* = 0.006), positive symptoms (*β* = −0.22, *p* = 0.001), negative symptoms (*β* = −0.36, *p* = 0.001), and general psychopathology (*β* = −0.098, *p* = 0.006) were inversely associated with overall quality of life of the respondents (Table 5).

## Discussion

This study was aimed to assess the overall quality of life profile with its domains and associated factors among people with schizophrenia and attending follow up service at Jimma University Medical Center, psychiatry clinic, Southwest Ethiopia. To the best of our knowledge, this study was the first study designed to examine the association between quality of life and psychiatric symptoms in Ethiopia, as the available study did not include psychiatric symptoms [17].

In this study, the mean of overall quality of life score among study participants was found to be (44.81 ± 8.87) and among the four domains of quality of life, respondents scored lowest mean in the social relationships domain (10.14 ± 3.12) which indicates that the social relationships domain of our study participants was the most affected. Previous studies done in Italy, India and Portuguese reported consistent results, showing that the social relationships domain of quality of life of people with schizophrenia was mostly affected [2, 25, 26]. This consistency of lowest score on the social relationship domain of QoL among people with schizophrenia could be due to the negative symptoms, which affect the patient’s ability to live independently, to perform activities of daily living, to be socially active and maintain personal relationships. Also, it could be due to the stigma towards mental illness, which isolates the mentally ill from social life [27].

In our study, regarding the socio-demographic variables, being divorced (*β* = −0.72, *p* = 0.02) and having no formal education (*β* = −0.69, *p* = 0.001) was significantly inversely associated with physical health domain of quality of life among people with schizophrenia. This result is inline to the previous study in which marital status and educational status were significantly associated with quality of life [17, 28, 29] and also inline to the study which reported that people with schizophrenia those with a low level of education report better quality of life [13]. Different studies reported different results regarding the association between educational status and quality of life. People with schizophrenia who are unable to read and write are more likely to have a poor quality of life compared to those who are degree and above [17]. Other studies have also revealed an association between low educational status in schizophrenia and quality of life, whereby a better educational level was associated with better psychopathological status in the disease evolution [29]. There was also a study conducted in Portuguese which revealed that there is no significant association between quality of life and respondents’ marital status and educational status [26, 30].

This study result showed that being rural resident was associated inversely with physical health domain (*β* = −0.48, *p* = 0.01), with environmental domain (*β* = −0.64, *p* = 0.03), with social relationships domain (*β* = −0.45, *p* = 0.04), and with overall quality of life (*β* = −1.93, *p* = 0.006) of people with schizophrenia and this result was inline to the previous population-based study in Brazil [31]. This could be justified by different possibilities like individuals are more physically active in a rural area, whereas insufficiently active or sedentary subjects prevail in urban areas [32]. It has also been shown that social networks in urban environments are smaller than in rural areas and that the most significant aspects were frequent contact with relatives, identity with environment and the feeling of belonging to a social group, as the rural people live mostly in settlements or communities, indirect and daily contact with their relatives [33].

In this study also the respondents’ age has a positive association with the psychological domain (*β* = −0.371, *p* = 0.071) of quality of life and this result was in line with the previous study [34]. However, this report was inconsistent with a study done in Saudi Arabia which reported that there is a negative association between age and quality of life among people with schizophrenia [35]. This discrepancy could be due to the difference of analysis method as the study in Saudi Arabia used Pearson or Spearman's product-moment correlation test to examine the association between QoL and independent variables, however, in the current study we used multiple regression analysis.

In our study, there was no significant association between quality of life and sex. In contrary to this result, in the previous studies, respondents’ sex was found having a significant association with quality of life [28, 35]. This difference could be due to the different instrument used to detect quality of life as the previous study [35] used Schizophrenia quality of life (S-QoL) which has 18 items measuring eight dimensions: psychological well-being (PsPhW), self-esteem, family relationship, relationship with friends, resilience, physical well-being, autonomy, and sentimental life, each scoring from 1 to 5. And also, this study used Pearson and Spearman's coefficient to detect the association between quality of life and socio-demographic variables, but in the current study, we used the WHOQOL BREF tool and multiple regression analysis.

In our study regarding the psychiatric symptoms, multiple regression analyses revealed a statistically significant negative association between psychiatric symptoms and quality of life. This study shows that positive psychiatric symptoms and negative symptoms were inversely associated with the physical health domain, psychological domain, and social relationships domains of quality of life and this result was supported by previous study results [16, 25]. However, another previous study finding revealed that the psychological health domain of quality of life was negatively associated with positive and general psychopathology symptoms but not significantly associated with negative symptoms[25].

Our study also revealed that the environmental health domain of quality of life had significant negative association with negative symptoms (*β* = −0.09, *p* = 0.001) and general psychopathology (*β* = −0.07, *p* = 0.001) and the important finding in the present study was, overall quality of life among people with schizophrenia had statistically significant negative association with positive symptoms (*β* = −0.22, *p* = 0.001), negative symptoms (*β* = −0.36, *p* = 0.001) and general psychopathology (*β* = −0.098, *p* = 0.006). This finding was in line with many previous study results [2, 15, 25]. This result indicates that psychiatric symptoms including positive, negative and general psychopathology were found to be key significant associated factors of poor quality of life among people with schizophrenia which indicates that utilizing comprehensive interventions to control all these psychiatric symptoms is mandatory to improve the quality of life of people with schizophrenia.

This study has some limitations. First, we used a self-reported method to assess QoL which may lead to a lack of objective measurements and an over-reporting of QoL. Second, due to its cross-sectional nature, the current study could not explore the cause and effect relationship of variables. Third, the number of variables examined in the study were relatively limited. Fourth, the study only recruited outpatients with schizophrenia who were actively attending their follow up service at the hospital and did not include people with schizophrenia who are living with their families in the community and inpatients, precluding generalization to all people with schizophrenia. Finally, the potential limitation of this study was the use of WHOQOL BREF, a generic questionnaire that may not have been sensitive enough to detect subtle changes in quality of life among people with schizophrenia.

## Conclusions

In this study, among domains of quality of life, the social relationship domain has the lowest mean score. Marital status, educational status, residence, and age were significantly associated with different domains of quality of life. Positive psychiatric symptoms, negative symptoms, and general psychopathology have a significant negative association with the overall quality of life. Therefore, priority interventions to improve the social deficits and comprehensive interventions that could address psychiatric symptoms among people with schizophrenia is essential to improve their quality of life.

## Supporting information

S1 File (SAV)
